# Iron Overload Is Associated With Oxidative Stress and Nutritional Immunity During Viral Infection in Fish

**DOI:** 10.3389/fimmu.2018.01296

**Published:** 2018-06-05

**Authors:** Estefanía Tarifeño-Saldivia, Andrea Aguilar, David Contreras, Luis Mercado, Byron Morales-Lange, Katherine Márquez, Adolfo Henríquez, Camila Riquelme-Vidal, Sebastian Boltana

**Affiliations:** ^1^Department of Biochemistry and Molecular Biology, Faculty of Biological Sciences, University of Concepcion, Concepción, Chile; ^2^Interdisciplinary Center for Aquaculture Research (INCAR), Department of Oceanography, Biotechnology Center, University of Concepción, Concepción, Chile; ^3^Renewable Resources Laboratory, Biotechnology Center, University of Concepción, University Campus, Concepción, Chile; ^4^Grupo de Marcadores Inmunológicos, Instituto de Biología, Facultad de Ciencias, Pontificia Universidad Católica de Valparaíso, Valparaíso, Chile

**Keywords:** iron overload, electronic paramagnetic resonance spectroscopy, nutritional immunity, oxidative stress, infectious pancreaticc necrosis virus, RNA-seq

## Abstract

Iron is a trace element, essential to support life due to its inherent ability to exchange electrons with a variety of molecules. The use of iron as a cofactor in basic metabolic pathways is essential to both pathogenic microorganisms and their hosts. During evolution, the shared requirement of micro- and macro-organisms for this important nutrient has shaped the pathogen–host relationship. Infectious pancreatic necrosis virus (IPNv) affects salmonids constituting a sanitary problem for this industry as it has an important impact on post-smolt survival. While immune modulation induced by IPNv infection has been widely characterized on *Salmo salar*, viral impact on iron host metabolism has not yet been elucidated. In the present work, we evaluate short-term effect of IPNv on several infected tissues from *Salmo salar*. We observed that IPNv displayed high tropism to headkidney, which directly correlates with a rise in oxidative stress and antiviral responses. Transcriptional profiling on headkidney showed a massive modulation of gene expression, from which biological pathways involved with iron metabolism were remarkable. Our findings suggest that IPNv infection increase oxidative stress on headkidney as a consequence of iron overload induced by a massive upregulation of genes involved in iron metabolism.

## Introduction

Iron is a functional constituent of proteins involved in a wide range of biological process including oxygen transport, energy production, and DNA synthesis, becoming essential for nearly all organisms (1, 2). Despite being so important, it has toxic properties when presented in its free forms (3). Iron exists in two oxidation states, Fe^2+^ and Fe^3+^, which electron transfer may elicit the production of reactive oxygen species (ROS) responsible for tissue damage (2, 4). To avoid these negative effects, iron is available coupled with proteins such as transferrin (*tf*), lactoferrin (*lf*), ferritin, and hemoproteins as haemo- or haptoglobin (2, 5, 6). Iron absorption is either performed from the diet (as heme or free-iron) or efficiently recycled from senescent circulating erythrocytes. Consequently, iron absorption must be highly regulated to avoid toxic overloads (3). In vertebrates, absorption is mainly regulated by Hepcidin, a liver-derived hormone, which binds to the only know efflux transporter ferroportin (3, 7). Once bound, hepcidin induces transporter internalization and degradation, inhibiting release of iron to the bloodstream (8, 9). Hepcidin gene transcription is regulated by iron levels, a rise on the iron level in plasma induce upregulation hepcidin mRNAs (10), in contrast, when iron level are restricted expression of hepcidin is inhibited and, therefore, iron is released from tissues (11).

Several pathogens including virus, bacteria, fungi, and protozoa use different host-cell elements as niches for survival, where access to fundamental nutrients such as iron is an important driving force in to live. Evidence has recognized a crucial role in iron regulation involving host defense mechanisms, where iron deficit confers relative resistance to infection (7, 12–15). This lead to the concept of nutritional immunity, as a whole of constitutive and inducible mechanisms that regulate the iron availability to pathogens and thus limit their capacity to infect the host (1, 16). The iron sequestration seems to have a dual function of denying iron to invading microorganisms and protecting the host tissues from oxidative stress owing to Fenton chemistry. Not surprisingly, many microorganisms have evolved mechanisms that evade or subvert iron-targeted nutritional immunity (17). For example, many viruses disrupt iron homeostasis inducing increase on iron intracellular loads and leading the viral disease develops (18). Indeed, viral replication increases the cell metabolism by using the cellular means to synthesize their own proteins (19, 20). Viral protein synthesis and genome replication require iron, becoming this mineral fundamental for efficient propagation on the host (21). In aquatic environments, the iron fertilization leads to blooms of phytoplankton (22), and consequently, as the blooms rates increase, viral replication should be boosted (23). Thus, viruses might be benefited from the increase of biological productivity that complements iron-induced cellular growth. However, as algal growth increase so does the replication of viruses infecting the organisms sharing the same habitat. Then, nutritional immunity should be a strong adaptive response protecting species living in high iron environments as observed in seas (18).

Infectious pancreatic necrosis virus (IPNv) is a bisegmented double-stranded RNA virus belonging to the family of Birnaviridae (24, 25). IPNv has an important impact on salmonids post-smolt survival, being one of the top three causes of losses in the salmon industry (26). Several studies have been performed aiming to understand the molecular mechanism of immune responses triggered by IPNv on salmonids. From them, it has been elucidated that *Salmo salar* infected with IPNv modulate immune responses, cytokine activity, stress response, metabolism, and hormone activity (26). Transcriptomic profiling comparing susceptible and resistant families have shown that a moderate but constant immune response including upregulation of genes involved with M2 macrophages system while downregulation of genes related to tissue differentiation and protein degradation confers protection to viral infection (27, 28). However, the mechanism by which IPNv outbreaks generates high mortalities on salmonids has not been completely elucidated. Through cellular culture, it has been shown that iron depletion, by hepcidin overexpression, reduces the infective capacity of IPNv (29). Furthermore, Atlantic salmon infected by *Piscine orthoreovirus* stimulate heme synthesis and iron metabolism (30), suggesting iron modulation induced by viral infection in fish. Up to date, no studies on fish have shown the impact of IPNv infection on iron metabolism and its further interactions with the cellular function. In the present work, we evaluated short-term effect of IPNv on several infected tissues from *Salmo salar*. We observed that IPNv displayed high tropism to headkidney (HK), which was correlated with an increase in iron load and oxidative stress responses in HK. Further, transcriptional profiling on headkidney showed a massive modulation of iron metabolism-related genes such as a strong modulation of hepcidin, ferritin, or ferroportin suggesting a key role of the nutritional immunity during a viral infection in fish.

## Materials and Methods

### Virus Isolation and Quantification

Chilean IPNv was isolated from *Salmo salar* headkidney by tissue homogenization on PBS and posterior centrifugation. The infectious supernatant was used to infect CHSE-214 cell line for viral isolation, plaque cloning, and subsequent passage by sequential transfer in cell culture. Monolayer cultures of CHSE-214 cells were maintained in Eagle’s minimum essential medium (EMEM) containing 10% fetal bovine serum. For virus amplification, drained monolayer cultures were infected at a level of infection (multiplicity of infection) of 0.01 plaque forming units (PFU) per cell. Viral adsorption was allowed during 1 h at 15°C to posteriorly add EMEM 5% fetal bovine serum. Viral detection was examined by qPCR with primers (WB117) and Universal ProbeLibrary probes (UPL) specific for the VP2 segment of the IPN virus.

### Animals and Culture Conditions

The experiments were performed at the ThermoFish Lab, Biotechnology Center, University of Concepcion, Concepcion, Chile. All experimental procedures were carried out in compliance with “International Guiding Principles for Biomedical Research Involving Animals” established by the European Union Council (2010/63/EU). *Salmo salar* at eggs stage were obtained from AquaGen S.A., Melipeuco, Chile, and were maintained on tanks with recirculating freshwater, flow rate of 5 m^3^ h^−1^, and water was U.V.-sterilized. A 24 h dark cycle photoperiod was used until the embryos hatched and cultivation parameters were controlled, water temperature (7 ± 0.7°C), dissolved oxygen (9 mg L^−1^), total ammonia concentrations (0.05 mg L^−1^), nitrite concentrations (0.01 mg L^−1^), and pH (8.0 ± 0.5) during this period. Once the yolk sac was completely absorbed, the photoperiod was changed to 12:12-h light-dark photoperiod (L:D), water temperature was gradually increased to research 12°C (±0.8°C) and they were fed twice a day with a commercial diet (BioMar).

### Experimental Design and Sampling

Parr Atlantic salmon, *Salmo salar* (121 ± 11.3 mg) were used for the viral challenge (*n* = 30). A total of twenty fish were selected for control and challenge group. Fish were starved for 12 h and then challenged by immersion method (31) in 5 L water with a dose of 10 × 10^5^ PFU/mL^−1^ of clarified supernatant from IPNv-infected CHSE-214 cell monolayers (*n* = 15). In parallel, fish in a control tank was similarly treated by adding 100 mL of virus-free cell culture supernatant to the water (*n* = 15). The fish were kept in the bath for 2 h and then was separated into two different tanks with the same original conditions. Fish were maintained for 24 h in these tanks. The order of sampling was decided randomly. For sampling 100 mg mL^−1^ MS-222^®^, Tricaína methanesulphonate (Sigma-Aldrich, MO, USA) was used to partially sedate fish decreasing stress during sampling. Blood was extracted from *vena caudalis* and kept on ice in heparinized tubes until centrifuged to separate the plasma, which was then snap-frozen in liquid nitrogen. Headkidney and liver samples of each individual group were dissected and immediately frozen in cryotubes in liquid nitrogen. All samples were stored at −80°C before RNA extraction.

### Indirect ELISA of Hepcidin, Cathelicidin-1

Plasma blood was used to determine the presence of Hepcidin and Cathelicidin-1 (32), through indirect ELISA (*n* = 10 fish by treatment, control, and virus challenge). Briefly, each plasma sample was worked in duplicate and diluted in carbonate buffer (60 mM NaHCO_3_, pH 9.6) to 35 ng/µL (100 µL). Briefly, each plasma sample was diluted in carbonate buffer (60 mM NaHCO_3_, pH 9.6), planted (in duplicated for each marker) at 35 ng/µL (100 µL) in a Maxisorp plate (Nunc, Thermo Fisher Scientific, Waltham, United States), and incubated overnight at 4°C. After, each well was blocked with 1% bovine serum albumin (BSA) for 2 h at 37°C. Then, plates were incubated for 90 min at 37°C with the primary antibody anti-synthetic epitope (diluted en BSA) of Hepcidin (diluted 1:500) and Cathelicidin-1 (diluted 1:500). Later, the second antibody-HRP (Thermo Fisher Scientific, Waltham, MA, United States) was incubated for 60 min at 37°C in 1:7,000 dilution. Finally, 100 µL per well of chromagen substrate 3,3′,5,5′-tetramethylbenzidine single solution (Invitrogen, CA, United States) was added and incubated for 30 min at room temperature. Reaction was stopped with 50 µL of 1 N sulfuric acid and read at 450 nm on a VERSAmax microplate reader. All assays were performed in triplicate. In the case of indirect ELISA, a calibration curve was used to evaluate the antibody efficiency. Briefly, 100 mL of each peptide with concentrations of up to 31.25 ng mL^−1^ were incubated overnight at 4°C and then washed with PBST 0.05% in a Mindray Microplate washer.

### Erythrocytes Separation

Erythrocytes fraction was obtained from blood using a discontinuous gradient of Lymphocyte Separation Medium (LSM, Corning, NY, USA) according to the protocol described by Peterson and colleagues (33). Briefly, blood was diluted 1:4 in 0.01 M PBS, pH7.4 (Gibco, Thermo Fisher Scientific, MA, USA) and loaded on a gradient containing 4 mL of LMS with a density of 1.075 g mL^−1^ overlaid with 3 mL of LMS with a density of 1.060 g mL^−1^. The gradient contained 0.19 M NaCl (pH 7.3). After centrifugation at 400 *g* per 30 min, the leukocyte fraction was collected from the 1.075 g mL^−1^ density layer and the 1.075–1.060 g mL^−1^ interface of LSM gradient. The leukocyte fraction was washed by mixing with PBS and frozen at −80°C. Erythrocytes obtained were posteriorly used for RNA extraction (*n* = 10 fish by treatment, control, and virus challenge).

### RNA Extraction and cDNA Synthesis

Total RNA was extracted from headkidney, liver, and red blood cells (RBC) with TRI Reagent^®^ (0.5 mL; Sigma-Aldrich, MO, USA) according to manufacturer’s instructions (*n* = 10 fish by treatment, control, and virus challenge). The concentration of RNA was assessed with the NanoDrop^®^ ND-1000 UV-Vis Spectrophotometer (Thermo Scientific, MA, USA), a 260/280 nm absorbance ratio of 1.8–2.0 indicates a pure RNA sample. RNA integrity was analyzed by denaturing gel electrophoresis. cDNA was synthesized from total RNA (200 ng/µL) using the RevertAid H Minus First Strand cDNA Synthesis Kit (Fermentas, Waltham, MA, USA) according to the manufacturer’s indications. RNA was stored a −80°C.

### High-Throughput Transcriptome Sequencing: Library Construction and Illumina Sequencing

Nine individuals from each group were selected for total RNA extraction, using a part of headkidney (20 mg), RNA extraction was individually isolated using Ribo-Pure™ Kit (Ambion^®^, USA) according to the manufacturer’s instructions. The RNA obtained was subsequently treated with DNase I (Fermentas, MA, USA) to remove genomic DNA according to the manufacturer’s protocol. RNA integrity number (RIN) was evaluated through the 2200 TapeStation (Agilent Technologies, CA, USA) using the R6K screen tape and reagents (Agilent Technologies, CA, USA). Samples with RIN values ≥ 8 and 260/280 ratio ≥ 1.8 were used for library construction. Total RNA from three individuals by each condition were pooled and quantified with Qubit^®^ 2.0 Fluorometer (Invitrogen, CA, USA) (*n* = 3 pools by treatment). Samples were prepared for Illumina sequencing using KAPA Stranded mRNA-Seq Kit (KapaBiosystems, MA, USA) according to the manufacturer’s instruction. Libraries were analyzed on the 2200 TapeStation (Agilent technologies, CA, USA) using D1000 screen tape and reagents (Agilent Technologies, CA, USA) and quantified by qPCR using the Library Quantification Kit Illumina/Universal (KapaBiosystems, MA, USA) according to the manufacturer’s instructions before pooling for sequencing on MiSeq (Illumina, Inc., CA, USA) platform using a run of 2 × 250 paired-end reads at the Laboratory of Biotechnology and Aquatic Genomics, Interdisciplinary Center for Aquaculture Research (INCAR), Universidad de Concepción, Chile.

### RNA-seq Data Analysis

Raw reads for both conditions were mapped to the genomic annotation for *Salmo salar* (release 100, ICSASG_version 2, NCBI) using Tophat2 software (34). Per condition mapped reads were assembled into transcripts using as reference the transcriptome annotation of *Salmo salar* through the implementation of Cufflinks2 package (35). In order to consolidate transcriptome assembly, we used the package Cuffmerge and posterior transcript quantification was performed with Cuffquant obtaining per condition expression normalized in FPKM values (Fragments Per Kilobase Million). Differential expression analyses were performed using Cuffdiff package, statistical analyses were done using the statistical model incorporated on this package (35) considering a gene differentially expressed with FDR < 0.05.

### Relative and Absolute mRNA Quantification

qPCR reactions were performed using the Maxima SYBR Green qPCR Master Mix (2X) (Fermentas). Each qPCR mixture contained the SYBR Green Master Mix, 2 µL cDNA, 500 nmol/L each primer and RNase free water to a final volume of 10 µL. Amplification was performed in triplicate on 96-well plates with the following thermal cycling conditions: initial activation for 10 min at 95°C, followed by 40 cycles of 15 s at 95°C, 30 s at 60°C, and 30 s at 72°C. The list of primers used in this study is included in Table S1 in Supplementary Material. An absolute quantification approach was used that involved calculating the number of gene copies in unknown “test” samples from comparison with a standard curve prepared using a serial dilution of linearized plasmids with known concentrations (36). The PCR product for each gene was extracted from agarose gel using the Nucleospin Gel and PCR Clean-Up Kit (MACHEREY-NAGEL, Dueren, Germany). The PCR amplicons were cloned the using pGEM-T Easy Vector and JM109 High-Efficiency Competent Cells (Promega, Madison, WI, USA). The Nucleospin Plasmid Quick Pure Kit (MACHEREY-NAGEL) was used to purify the plasmid DNA containing the PCR insert. Then, the plasmid was linearized using the HindIII restriction enzyme to prevent amplification efficiency problems that can arise from using supercoiled plasmids, and the amount of dsDNA was quantified using the QuantiT PicoGreen dsDNA Assay Kit (Invitrogen, CA, USA). Copy number calculations, using the following equation, were performed based on the concentration of each plasmid, obtained by absorbance at 260 nm, and the values observed from the five-fold serial dilution produced by qPCR:

$$N_{\text{copy}}=\frac{\text{amount}\times 6.022\times 10^{23}}{\text{length}\times 1\times 10^{9}\times 650}.$$

The use of these standard curves controlled for amplification efficiency differences between assays and allow the calculation of “absolute” number of mRNA transcripts, thereby facilitating gene comparisons (*n* = 10 fish by treatment).

### Electronic Paramagnetic Resonance (EPR) Analysis (EPR Spin Trapping)

In order to identify free radical species generated in oxidative processes, promoted by the infection, EPR analysis was carried out with an EPR EMX micro 6/1 Bruker spectrometer (*n* = 10 fish by treatment). EPR equipment works in the band X equipped with a Bruker Super High QE resonator cavity and 5,5-dimethyl-1-pyrroline N-oxide (DMPO) as a spin trap. For this analysis, 20 mg of liver and 20 mg kidney tissue from fish no- and infected with IPN virus were macerated in a solution of DMPO 100 mmol L^−1^ in dimethylsulfoxide (DMSO). The measurements were made in a flat EPR cell at laboratory temperature (19°C). The instrumental conditions were: field center, 3514 G; sweep width, 200 G; microwave power, 20 dB; frequency, 100 kHz; constant time 0.01 ms; weep time, 30 s; amplitude modulation, 1.00 G; and receiver gain, 30 dB. 50 spectral accumulations were made for each of the samples. The calibration curve was carried out using 4-hydroxy-2,2,6,6-tetramethylpiperidine-1-oxyl (TEMPOL). The identification of the radical species produced was based on the comparison of the coupling constants a_N_ and a_H_ measured in the adducts formed with those reported in the literature for the DMPO/^⋅^OH adduct in DMSO. The simulation and adjustment of the EPR spectra were performed using Bruker’s Xenon software.

### Statistical Analyses

The viral-challenge analysis was carried out by pairwise comparisons between groups using the Bonferroni method for adjusting the level of significance. Predictions of fish numbers from final models were calculated and plotted to evaluate interactions. All statistical analyses were performed with Stata version 14 (StataCorp LP). For gene expression, absolute mRNA quantification by qPCR, the data were tested for normality and homogeneity of variances using the Shapiro–Wilk’s and Levene’s test respectively. When necessary, expression was log_10_ transformed to achieve normality and all variances were homogeneous. Data obtained was analyzed using Statistica 6.0 software (Statsoft Inc., Tulsa, OK, USA) and two-way ANOVA followed by Tukey HSD *post hoc* test for multiple comparisons. Results were considered significant when *p* < 0.05. Graphs were plotted with GraphPad PRISM v6.0 (GraphPad Software, Inc., CA, USA).

## Results

### High Tropism of IPNv to Headkidney Dramatically Increase the Oxidative Stress in This Tissue

**Figure 1:**
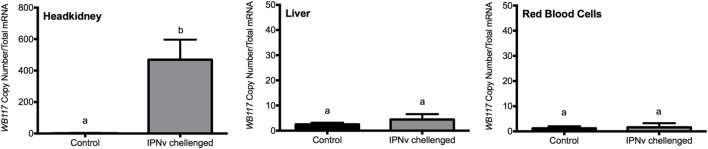
Viral load expressed in terms of VP2 segment abundance. Measured by qPCR from headkidney, liver, and red blood cells. The experimental groups are: virus free (control, black bar), and infectious pancreaticc necrosis virus challenged (gray bar). Different letters denote significant differences between groups. Values are represented as the mean VP2 copy number ± SD. Different letters denote significant differences between experimental groups (two-way ANOVA; *p* < 0.05; *p* < 0.0001).

In order to quantify viral load on different tissues infected with IPNv, tissue samples from headkidney, liver, and RBC were analyzed. Total RNA was isolated at 24 h after immersion challenge (hpc) with IPNv or virus-free cell culture supernatant (control). IPNv detection was performed by qRT-PCR using a Universal ProbeLibrary (UPL) specific for the VP2 segment of IPNv. After infection, the estimation of the viral copy number showed higher loads on headkidney (6 × 10^2^), followed by liver (1 × 10^1^), and almost undetectable in RBC. This result indicates that IPNv is capable of infected more than 60-folds greater the headkidney than the liver (Figure 1) after only 24 post-infection.

**Figure 2:**
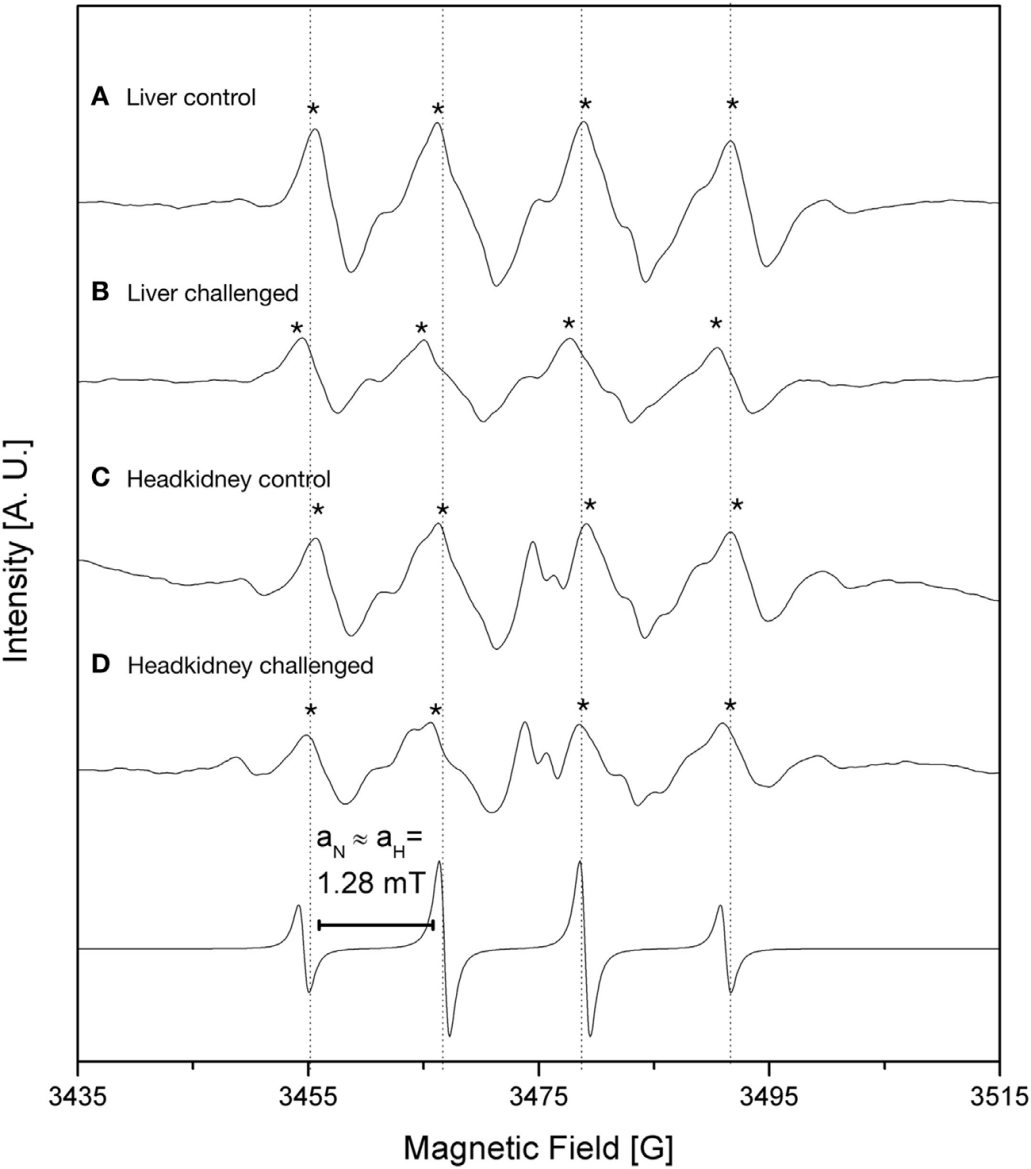
Electronic paramagnetic resonance spectra of DMPO spin adducts in fish tissues challenged with infectious pancreaticc necrosis virus (IPNv) virus (53.3 mM, pH 3.6 with 1 N NaOH). **(A)** liver control, **(B)** liver IPNv challenged, **(C)** head kidney control, **(D)** head kidney IPNv challenged. Upper and lower traces are experimental and simulated spectra, respectively. DMPO/^⋅^OH spin adducts are denoted (*).

The iron load was quantified indirectly through the detection of oxidative stress reactive species. We inspect the levels of free radicals generated by oxidative processes through EPR analysis using DMPO as a spin trap. The ROS, such as ^⋅^OH, are triggered by free iron which is quickly converted into oxidative species through the Fenton reaction. We measured radical ^⋅^OH species in liver and headkidney, in both IPNv-infected and non-infected fish (Figure 2; Table 1). To quantify the production of radical ^⋅^OH, we used interpolation of a TEMPOL calibration curve (see methods). As shown in Table 1, both non-infected liver and headkidney display similar amount of free radicals (1.43 × 10^−6^ mol L^−1^ in liver and 1.57 × 10^−6^ mol L^−1^ in headkidney). On infected tissue, the production of radical ^⋅^OH is dramatically increased in headkidney (2.02 × 10^−6^ mol L^−1^) compared to liver displaying a slight increase (1.70 × 10^−6^ mol L^−1^). The present results show a high availability of iron in headkidney as a consequence of the ^⋅^OH levels observed in the tissue. The correlation between the RNA abundance of the viral segment VP2 (Figure 1) and iron load (Figure 2; Table 1) suggest that IPNv, in a short period, preferentially infects headkidney.

**Table 1:** Quantification of the DMPO/^⋅^OH adduct by electronic paramagnetic resonance (EPR) spectroscopy.

Samples	Concentration (mol L^**−**1^)	SD	Comparison	*p-*Value
Liver control (LC)	1.43 × 10^−6^	1.46 × 10^−8^	LC vs LI	***

Liver infectious pancreaticc necrosis virus (IPNv) (LI)	1.70 × 10^−6^	4.50 × 10^−8^	LI vs HI	***

Headkidney control (HC)	1.57 × 10^−6^	1.84 × 10^−8^	HC vs HI	***

Headkidney IPNv (HI)	2.02 × 10^−6^	7.94 × 10^−8^	LC vs HC	ns

EPR measurement of hydroxyl radicals (^⋅^OH) in headkindey and liver tissue. Data compares (mean ± SEM) amount of ^⋅^OH in arbitrary units. Headkindey and Liver were extracted and stabilized with spin trap DMPO. Asterisks present statistical analysis: one way ANOVA with Tukey’s post-doc test (see table), **p < 0.01, ***p < 0.001.

### Headkidney Display Antimicrobial Responses Soon After Infection

**Figure 3:**
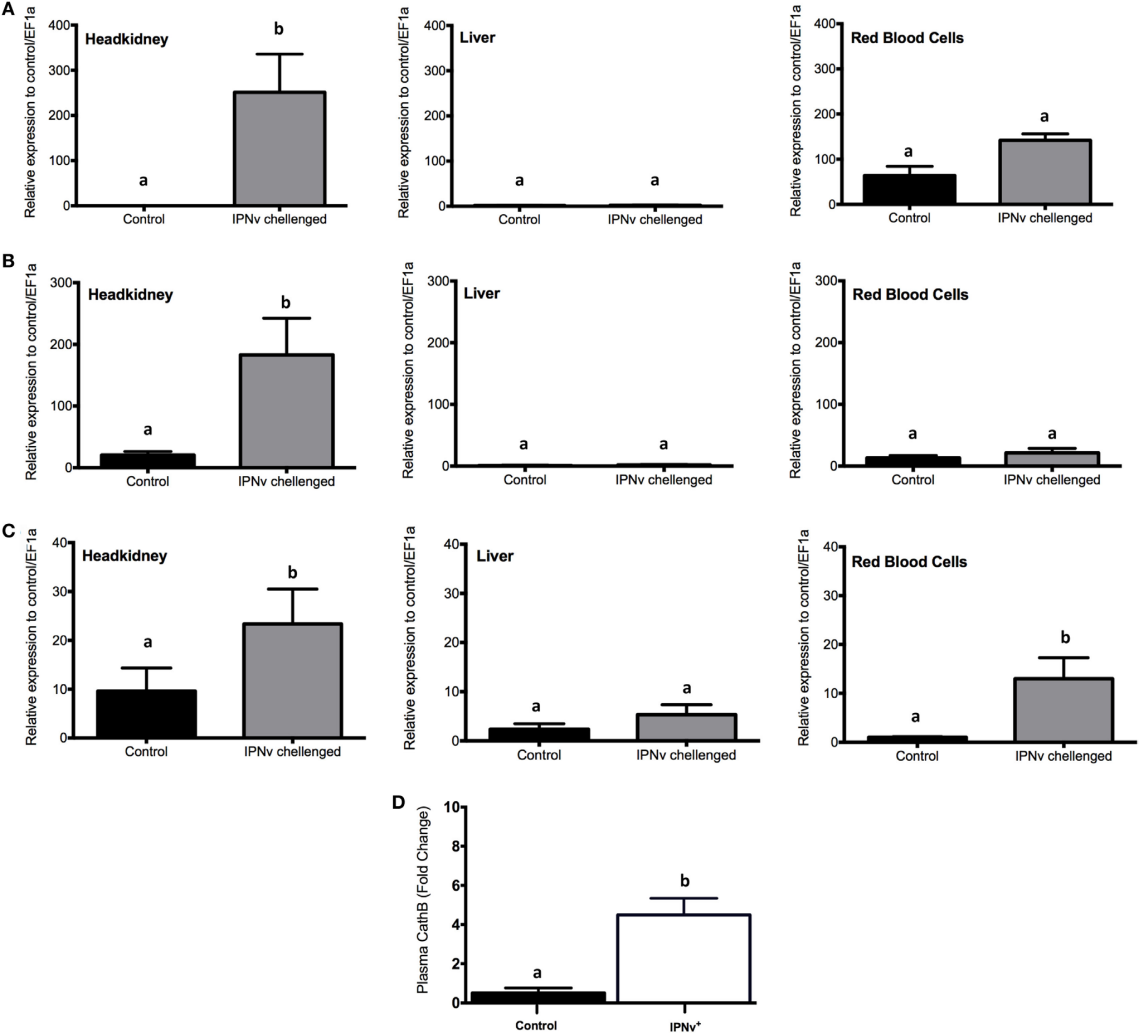
Expression profile of genes associated with antiviral responses. mRNA expression measured by qRT-PCR for **(A)** Mx, **(B)** IFN-y, and **(C)** cathelicidin from headkidney (left), Liver (middle), and red blood cells (right). **(D)** *Salmo salar* plasma concentration (pg × mL^−1^) of Cathelicidin. The experimental groups are: virus free (control, black bar), infectious pancreaticc necrosis virus challenged (gray bar). Values are represented as the mean mRNA/protein abundance ± SD. Different letters denote significant differences between groups (two-way ANOVA; *p* < 0.05; *p* < 0.0001).

In order to explore the activation of antiviral responses in different organs, we evaluated by qRT-PCR the expression of *Mx, INF-y*, and *cathelicidin* on infected and non-infected samples from headkidney, liver, and RBC (Figure 3). We observed that after 24 h post-challenge (hpc), the expression of *Mx, IFN-y*, and *cathelicidin* is highly upregulated in headkidney (Figures 3A–C). In contrast, the expression of these genes in liver and RBC remain unchanged with the exception of *cathelicidin* in RBC were upregulation was observed (Figure 3C). To confirm that mRNA upregulation of *cathelicidin* reflects a peripheral increase in this antimicrobial peptide, we measured the level of this peptide in blood plasma by ELISA assay. This analysis confirmed the significant increase of *cathelicidin* in blood plasma (Figure 3D). Then, our results indicate activation of antimicrobial responses in headkidney, which is triggered by the high infective rate of IPNv to this organ leading to the development of a fast host response.

### Global Gene Expression Changes Induced by IPNv in Headkidney

**Figure 4:**
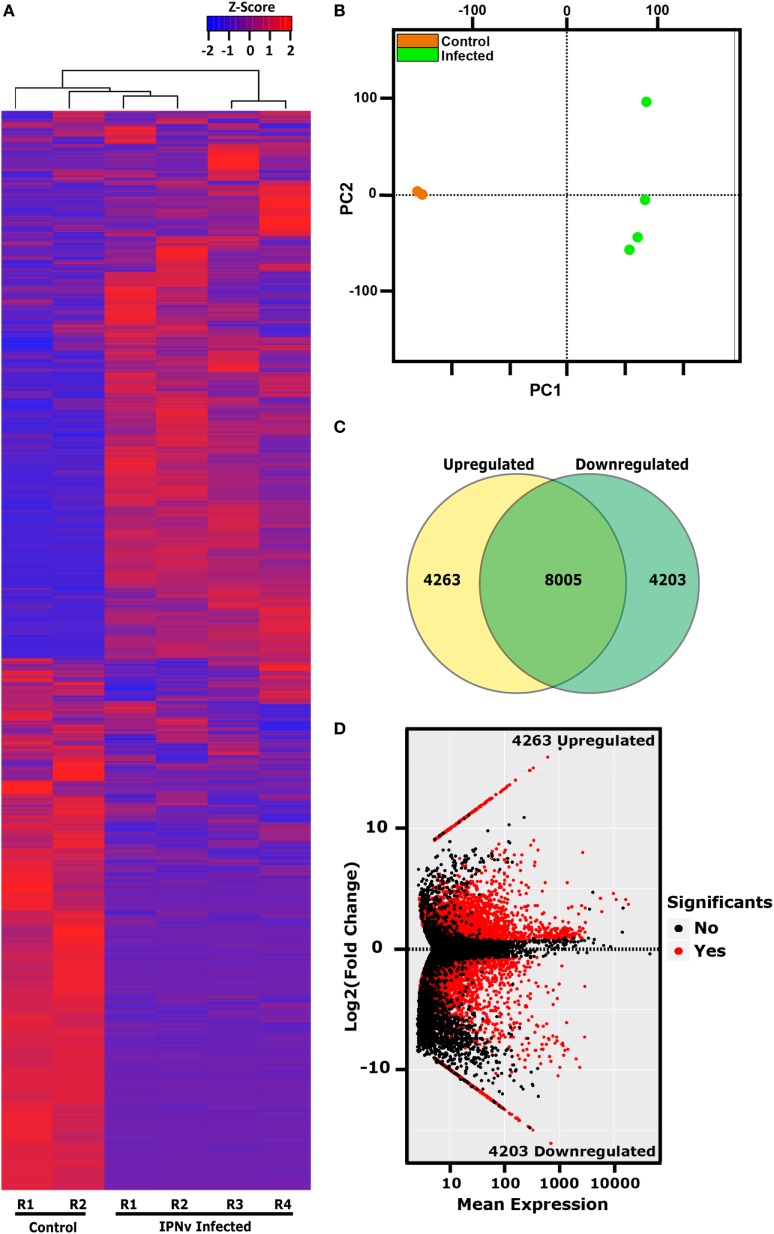
Transcriptional dynamics of coding genes in headkidney during infectious pancreaticc necrosis virus (IPNv) infection. **(A)** Gene expression clustering of the 16,470 genes expressed on headkidney. **(B)** Principal component plot (PCA) of sample replicates (orange = control samples and green = infected samples). **(C)** Venn diagram indicating the number of differentially expressed genes. **(D)** MAplot (average expression vs. fold of change) displaying all differentially expressed genes induced by IPNv infection.

**Figure 5:**
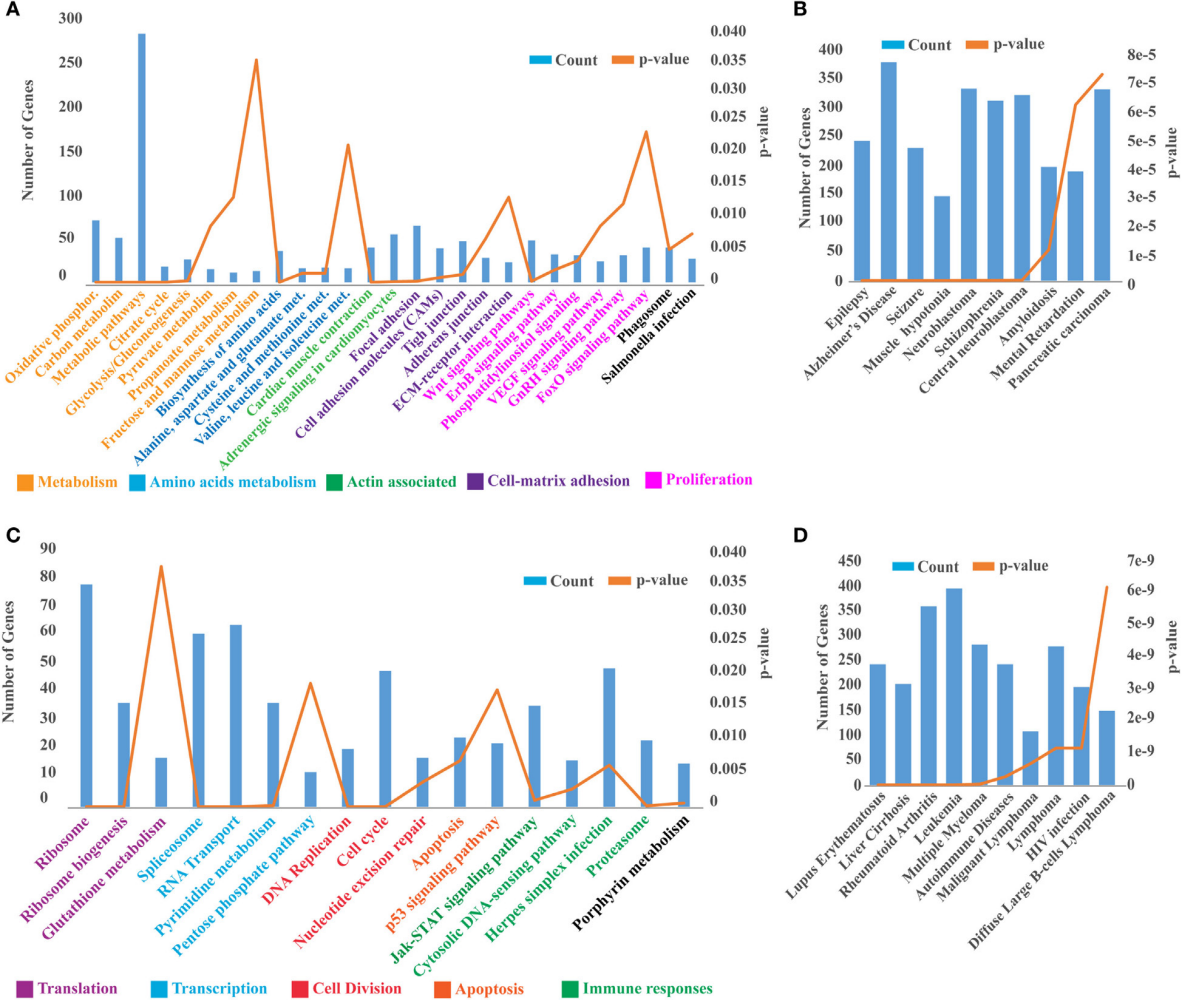
Functional annotation of pathways and disease modulated during infectious pancreaticc necrosis virus infection. **(A)** Functional enrichment analysis displaying the biological pathways enriched by the set of downregulated genes, **(C)** and upregulated genes. **(B)** Top 10 disease enriched by the set of downregulated genes, **(D)** and upregulated genes. Biological pathways were manually group and colored by biological process.

As our results indicate that headkidney is the most affected and responsive tissue at 24 hpc, we decided to explore the transcriptomic profile of headkidney during IPNv infection. Samples from infected and non-infected animals were sequenced and posteriorly analyzed identifying a total of 16,470 coding genes expressed on headkidney in either or both conditions. As shown in Figure 4A, a clear pattern of gene modulation was observed when clustering the expression of infected and non-infected samples on a heatmap plot. Same results were observed through principal component analysis, showing that replicates from the same condition form clusters on the first principal component (PC1), indicating high reproducibility of our preparations (Figure 4B). In order to identify modulated genes, which expression significantly changed, we performed a differential expression analysis and identified 8,465 genes modulated during IPNv infection (FDR < 0.05). From them, 4,263 genes were upregulated and 4,203 genes were downregulated as shown in Figures 4C,D. To have a global idea about biological pathways affected by IPNv infection, we performed a functional enrichment analysis for the set of upregulated and downregulated genes using KEGG pathways database and ToppFun server for disease enrichment. For downregulated genes, in Figure 5A, we observed a massive shutdown of biological pathways associated to metabolism such as oxidative phosphorylation, glycolysis, and citrate cycle driven by the downregulation of genes coding for enzymes such as *ATP synthases, NADH dehydrogenases, cytochrome c oxidases, succinate dehydrogenases, lactate dehydrogenase, pyruvate dehydrogenase, citrate synthase, fumarate*, and *succinate-CoA ligase subunits* between others (see Tables S2 and S3 in Supplementary Material). The expression of genes involved in amino acids metabolisms such as *asparagine synthetase, glutamate decarboxylase 1b and 2*, and *glutaminase a* were downregulated. Genes associated with actin filaments such as *tropomyosin 1-4a, alpha-tropomyosin, calmodulin*, and *troponin C type 1*, drove the downregulation of Cardiac muscle contraction and Adrenergic signaling in cardiomyocytes pathways. Additionally, biological process related to cell-matrix adhesion were attenuated such as focal adhesions, cell adhesion molecules, tight junctions, adherent junction, and ECM-receptor interaction (see Tables S2 and S3 in Supplementary Material). We also observed inhibition of pathways associated to tissue repair, proliferation, and migration such as Wnt, ErbB, GnRH, phosphatidylinositol, FoxO, and VEGF signaling pathway through the attenuation of MAPK, calcium, and PI3K/Akt signaling. Finally, the phagosome maturation and cellular response to salmonella infection pathway were also downregulated indicating a partial blockage of innate immune responses. In other hand, the disease enrichment analysis showed several terms associated to nervous system related diseases which are driven by the downregulation of genes associated to signaling (*thra, apc, nr1l2*), transport (*slc12a5, slc9a6, kcnab2, cacna1a, clcn4*, among others), structural proteins (*col1a1, col1a2, col2a1, tubb2a, tpm3*), and transcription factors (*sox5, sox9, sox11, zeb2, foxq1*), among other genes (Figure 5B).

For upregulated genes, as shown in Figure 5C, we observed an activation of biological pathways such as translation and transcription which are probably hijacked by the virus. For example, ribosomal proteins, enzymes responsible for rRNA modifications, ribosome maturation, and cytoplasmic export are dramatically upregulated (see Table S3 in Supplementary Material). As well mRNAs coding for components of the Spliceosome (U1, U2, U4/U6, U5, Prp19, and EJC/TREX complex), pyrimidine metabolism, and RNA transport (Nuclear pore complex including several nucleoporins). Associated with immune responses, we found activated the JAK–STAT signaling pathway, Cytosolic DNA-sensing, and herpes simplex infection pathway leading to the overexpression of *type I interferon, interferon regulatory factors*, and *interferon-induced proteins*. Moreover, proteins components of standard proteasome and immunoproteasome were also upregulated. Supporting these observations, the disease enrichment analysis showed several terms associated to inflammation and immune responses (Figure 5D) such as leukemia, autoimmune disease, malignant lymphoma, and HIV infection.

### IPNv Induce a Massive Upregulation of the Genetic Machinery Involved in Intracellular Iron Availability

**Figure 6:**
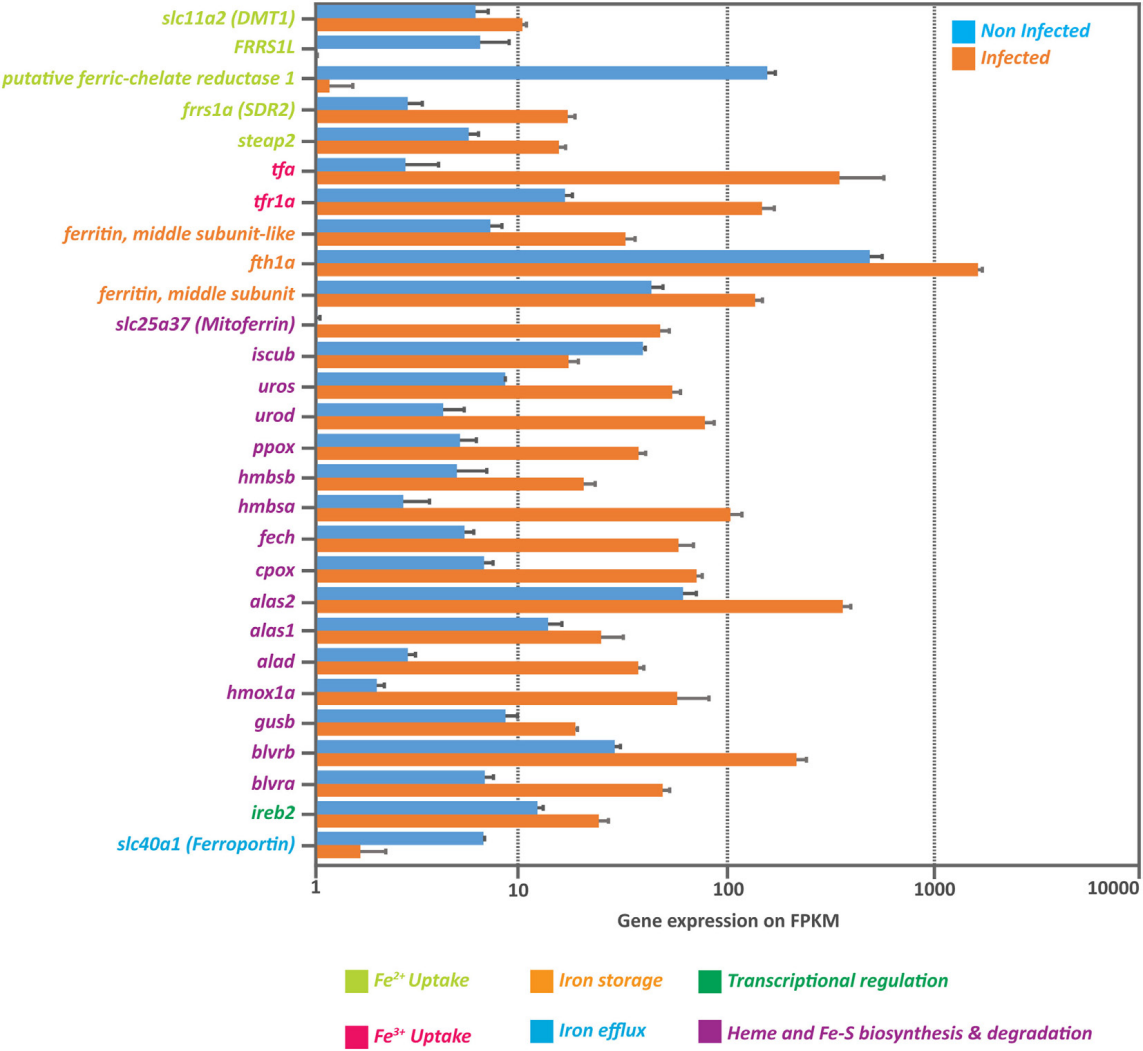
Massive upregulation of genes involved in iron intracellular availability. Expression of genes associated to iron uptake, storage, heme synthesis and degradation as well as transcriptional regulation, obtained from the RNA-seq data from infected and non-infected headkidney (normalized expression in Fragments Per Kilobase per Million reads or FPKM).

Our results indicated that IPNv efficiently infects headkidney, remarkably increased the production of free radicals as an indirect measure of high iron load, triggering a massive transcriptome dysregulation. It has been widely documented that viral infections induce oxidative stress in infected tissue through iron overload. Viruses induce cellular iron overload because fundamental processes such as genome replication and protein synthesis require this mineral. To evaluate if IPNv induces iron overload in headkidney from *Salmo salar*, we explored the expression of genes tightly linked with iron uptake, transport, and storage on our RNAseq data. As shown in Figure 6, we observed upregulation of genes associated to Fe^2+^ and/or Fe^3+^ uptakes such as iron reductases (*frrs1, steap2*), iron channel (*slc11a2*), transferrin (*tfa*), and transferrin receptor (*tfr1a*), which suggested a rise on intracellular iron. As iron uptake is potentiated, mechanisms related to the iron intracellular storage seems to be activated. We observed an increase in the mRNA abundance of ferritin (both middle and heavy subunits) and Heme synthesis process (*alad, alas1, alas2, cpox, fech, hmbsa, hmbsb, ppox, urod, uros*) as well as the mitoferrin transporter (*slc25a37*). In addition, we observed upregulation of genes associated with heme degradation (*blvra, blvrb, gusb, hmox1a*) that could help raising the levels of circulating iron. Finally, we observed that iron efflux seems to be attenuated as the expression of ferroportin (*slc40a1*) is downregulated. These results together suggest an increase in iron bioavailability that could be responsible for the rise in oxidative stress.

### Homeostatic Response to Iron Overload

**Figure 7:**
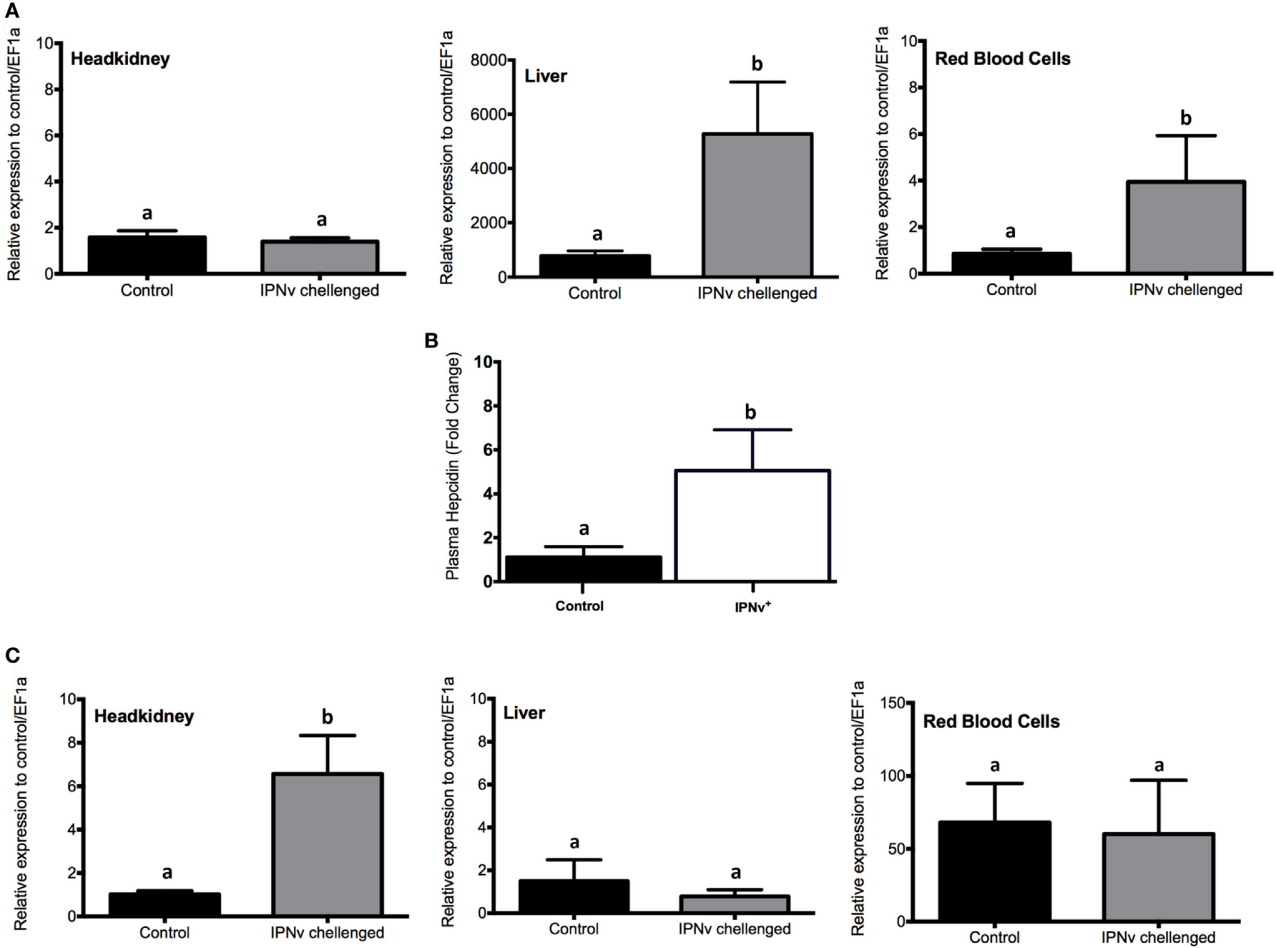
mRNA and protein expression profile of genes associated with the iron homeostatic response. Expression measured by qRT-PCR for **(A)** hepcidin, **(C)** heme oxygenase-1 from headkidney (left), Liver (middle), and red blood cells (right). **(B)** Plasma concentration (pg × mL^−1^) of Cathelicidin. The experimental groups are: virus free (Control, black bar), IPNv challenged (gray bar). Values are represented as the mean mRNA/protein abundance ± SD. Different letters denote significant differences between groups (two-way ANOVA; *p* < 0.05; *p* < 0.0001).

As the increase of oxidative stress might be explained by high levels of intracellular iron, we evaluated if homeostatic responses were activated in order to lower iron levels. Much progress has been made in our understanding of the role of the “classic” antioxidant enzymes (e.g., superoxide dismutase, catalase, glutathione peroxidase) in mediating of the oxidative stress. However, it is becoming clear that other oxidant-induced gene products may also play vital roles in the protective response to oxidative stress. One such stress-response protein the hepcidin and the heme oxygenase-1 (HO-1). By qRT-PCR, we assayed the expression of *hepcidin* and HO-1 on headkidney, liver, and RBC from infected and non-infected animals. As shown in Figure 7A, the expression of *hepcidin* increase in liver and RBC while no changes were observed in headkidney. We monitored peripheral levels of hepatic hepcidin in blood plasma by ELISA assay, which confirms the increase of this hormone in the plasma (Figure 7B). Moreover, the expression of HO-1 did not change in liver and RBC while it was upregulated on infected headkidney indicating a high rate of heme degradation on this organ (Figure 7C).

## Discussion

In the present work, we have evaluated the effect of IPNv infection on post-smolt *Salmon salar* after 24 h post-challenge. We measured viral load in headkidney, liver, and RBC by Absolute-qRT-PCR and observed that IPNv displayed higher infection rates for headkidney than for liver or RBC. High tropism of IPNv for headkidney, spleen, and pancreas was already described by Munang’andu and colleagues (37), however, the viral load on this study was evaluated only from 7 days post-challenge leading uncovered the early stages of infection in *Salmo salar*. Early detection of IPNv after immersion challenge has been documented on rainbow trout (24), the viral load observed in headkidney were detectable at 24 hpf (hours post fertilization). Additionally, the lower viral load in liver observed in our results are concordant with an early infection, as an increase in liver infection correlates with the onset of mortality (37).

Viral infection has a series of detriment effect on the host tissue, among them, oxidative stress is often associated with enhanced pathogenesis (38). In our study, higher viral loads in headkidney were accompanied by the rise in iron availability and free radical detection which were dramatically increased in comparison to the liver. In the same direction, our results showed that hepcidin upregulation correlates with lower viral loads in liver and RBC. It is possible that, at 24 hpc, the fast transcriptional response of liver and RBC could help decreasing iron intracellular availability, as a mean to eliminate/block IPNv on these tissues. However, further analysis including expression dynamics at long term are required to probe that hypothesis.

We observed that antiviral responses were also pronounced on headkidney, for instance, the expression of innate immune genes such as *inf-y, mx*, and cathelicidin was increased on infected samples and remains unchanged on liver and RBC. Interestingly, it has been documented that viral infection blocks the expression of Interferon as a strategy of immune evasion (39); however, it has also been reported that increase levels of Interferon correlate with viral replication (37), which is in agreement with our results. Experiments that have used cultured cells found that iron might increase the expression of virus replication genes, possibly through its effects on translation *via* eukaryotic translation initiation factor 3, eIF-3 (40, 41). Our results suggest that eIF-3 complex, which is required for several steps in the initiation of protein synthesis (42), also was highly modulated in headkidney of infected fish. In addition, the viral infection induce a strong transcriptional modulation in other transcriptional and DNA process mechanisms including RNA transport, spliceosome, nucleotide biosynthesis, DNA replication, and ribosome biosynthesis. In contrast, we observed a massive shutdown of pathways involved in energy and amino acid metabolism such as glycolysis, citrate cycle, and pyruvate metabolism were attenuated on infected headkidney. Our results are contrary to what has been described for widely studied viruses such as VIH-1 and HCMV, where a dramatic increase in glucose uptake, aerobic glycolysis, and pyruvate metabolism are essential for viral replication (43, 44). However, it has been described that infectious bursal disease virus or IBDv (a member of birnavirus family), infecting young chickens, downregulates the expression of proteins involved in energy metabolism as well as amino acids transport and metabolism (45) suggesting a family-specific metabolome modulation. On one hand, viral replication is highly dependent on nucleotide precursor such as the aspartate and glutamine amino acids (46–48), as they provide y-nitrogen for purine and pyrimidine biosynthesis (49). Our results showed downregulation of *asparagine synthase* and *glutaminase* lead to an increase of aspartate and glutamine, as well as an increase of pyrimidine metabolism and pentose phosphate pathways, which might be promoted by a rise in nucleotide precursors. In other hand, mitochondria is the main source of ROS through a combination of the mitochondrial electron transport chain and the Fenton reaction (50). Our results showed downregulation of several enzymes associated to glycolysis and the components of the mitochondrial electron transport chain, suggesting impaired oxidative phosphorylation leading to an increase in oxidative stress (51, 52). We also observed upregulation of the superoxide dismutase (*sod1* gene), a key enzyme for hydrogen peroxide (H_2_O_2_) formation (member of the Fenton reaction), supporting the contribution to increase oxidative stress. Then, based on our results, IPNv infection might increase oxidative stress on headkidney (in part) through mitochondrial dysfunction.

The present study show that in headkidney, IPNv induce the modulation of mRNAs tightly related with tissue degradation as extracellular matrix components (EMC) and cell–cell contact, which are required for successful viral replication and propagation (53). EMC is a complex structure involved in a wide range of biological processes such as cell proliferation, migration, and differentiation, which dysregulation leads to diverse pathologies (54). Our data also show that infected fish induces downregulation of several components of focal adhesion, EMC–receptor interaction, cell adhesion molecules, and other inflammatory cytokines in headkidney. The mentioned process suggests that the virus promote a tissue damage, which is follows for a leukocyte proliferation in the infected tissue. Interestingly, our previous data have identified a strong and significant increase of leukocyte immune effectors and tissue degradation in the headkidney of infected salmon (55). Our result support this observation because *il6, il1ß, il12, tnf-, ifny* mRNAs are significantly induced during infection, supporting the potential development of a specific leukocyte response. However, further studies are needed to support this hypothesis. Other birnavirus, as IBDv, also induces EMC damage affecting the organization of these molecules on highly susceptible cells in the spleen of chickens (56). Dengue virus for example is able to trigger the downregulation of host genes involved in maintaining cell junction integrity and collagen assembly (EMC) to accomplish successful infection (57). Then, ECMs might play a contrasting role during viral infection by supporting cell-to-cell viral transmission and restrict viral infection by a mechanism that still remains to elucidate.

A previous study have evaluated the transcriptional response of headkidney from IPN-resistant and susceptible families of Atlantic Salmon (28). The overall result of this work showed that IPN-susceptible families activate inflammatory responses soon after infection 1dpc, but they are not able to sustain this response in a long term (5dpc). Through a microarray assay, they compared transcriptional modulation induced by IPNv challenge and observed that IPNv induce upregulation of endocrine function, and downregulation of tissue proliferation, immune-related genes (mainly innate response), and protein metabolism. These results are in agreement with our observations at 1dpc concerning downregulated pathways such as amino acid metabolism, tissue proliferation and phagosome maturation, but we did not observed enrichment of pathways associated to the endocrine function. Our transcriptomic analysis uncovered modulation of iron-binding genes through the upregulation of heme synthesis and degradation suggesting the development of the nutritional immunity in response to the virus infection in fish. Based on our EPR results, we found an increase of free radical production on headkidney, which directly imply a large availability of iron in this tissue. Iron is indispensable for life, many proteins involved in crucial cellular processes require this mineral (58–60). Interestingly, viral infection induces an increase in iron bioavailability in host cells to promote virus replication and propagation (18). However, an excess of intracellular iron catalyze the generation of free radicals through Fenton Chemistry and produce damages on lipids, proteins, and DNA (18) leading to apoptotic processes. Further exploration of genes associated with oxidative stress, iron uptake, storage, and efflux on our transcriptomic data reflects an accumulation of intracellular iron. ROS may contribute to tissue damage in many pathophysiological conditions and participate in physiological signaling processes (61). In vertebrates, HO-1 has been widely studied as a model for redox-regulated gene expression (62). Furthermore, antioxidants and metal-chelating compounds can modulate HO-1 expression (63). The present data show an increase of the mRNA abundance *of heme oxygenase 1* in headkidney in individuals challenged and highlight the increase of the oxidative stress (ROS) in this tissue as a consequence of the IPNv infection. In addition, our study also show that the iron reductases, Fe^2+^ membrane transporters, transferring, and tfr1a responsible for Fe^3+^ internalization, ferritin for storage, mitochondrial iron transporter, and iron regulatory proteins where upregulated. Moreover, the iron efflux transporter, ferroportin (*slc40a1*), was downregulated supporting an increase in iron bioavailability and an increase of oxidative stress iron-mediated. Control of dietary iron absorption and systemic iron metabolism is mainly regulated by the hormone Hepcidin (3). Transcription of hepcidin gene is upregulated by high iron levels, infection and inflammation (64, 65), being released mainly by the liver and reducing iron export by binding to the iron exporter ferroportin-1 (3). We observed an overexpression of hepcidin mRNA in infected liver and RBC, as well as a rise in blood plasma levels in infected animals. These results suggest that IPNv increase the intracellular concentration of iron by a massive activation of genes involved in iron metabolism. This rise of intracellular iron, increase the production of free radicals and triggers homeostatic responses and might also promote apoptosis in infected headkidney.

In conclusion, our results highlight the close interaction between iron load, oxidative stress, and immune performance. Our data demonstrate the extensive plasticity of the immune response in fish and highlights the importance of iron homeostasis mechanisms where infected animals are able to develop an underlying nutritional immunity under viral infection. This approach delineates new immune mechanisms triggered during a viremia, where new vaccine strategies might be developed to improve the immunological performance and therefore increase the survival.

## Ethics Statement

All animal experiments conformed to international animal research regulations (the British Home Office Regulations. Animal Scientific Procedures Act 1986; care guidelines, EU 2010/63) and follow the guidelines for the use of laboratory animals established by the Chilean National Commission for Scientific and Technological Research (CONICYT), authorized by the Universidad de Concepcion Institutional Animal Care and Use Committee.

## Author Contributions

The study was conceived by SB with important input from ET-S and AA. CR-V, LM, BM-L, KM, and AH performed the experiments; ET-S and AA analyzed the data; SB and ET performed the model simulations and provided extensive additional input; SB funding acquisition; SB and ET-S drafted the manuscript with substantial contributions from all other authors.

## Conflict of Interest Statement

The authors declare that the research was conducted in the absence of any commercial or financial relationships that could be construed as a potential conflict of interest.

## Abbreviations

IPNv, infectious pancreaticc necrosis virus; tf, transferrin; lf, lactoferrin; EPR, electronic paramagnetic resonance; RBC, red blood cells; DMPO, 5,5-dimethyl-1-pyrroline N-oxide; DMSO, dimethylsulfoxide; TEMPOL, 4-hydroxy-2,2,6,6-tetramethylpiperidine-1-oxyl; hpc, hour post challenge; EMC, extracellular matrix components.
