# Supplementary material for: Iron Overload Is Associated With Oxidative Stress and Nutritional Immunity During Viral Infection in Fish
Source: Front Immunol. 2018 Jun 5;9:1296. doi: 10.3389/fimmu.2018.01296 (PMC5996096; doi:10.3389/fimmu.2018.01296)
Supplement: Supplementary file 1 [file table_1.docx]

| **Gene** | **Name** | **Primer 5'-3'** | **Tamaño (bp)** | **E**  **(%)** | **Tm**  **(°C)** | **GenBank accession n°** |
| --- | --- | --- | --- | --- | --- | --- |
| ALA_d | Delta-Aminolevulinic Acid Dehydratase | F:CCACTCGCCCATCCATCATA | 150 | 91.5 | 60 | XM_014128803.1 |
|  |  | R:ACACCTCACATGGACACTGT |  |  |  |  |
| ALA_s | Aminolevulinate, Delta- Synthase 2 | F:TCCAGCAGCACATGGAAGTT | 150 | 86.48 | 62 | XM_014146047.1 |
|  |  | R:GGTGTCTGTCTGGTGCAGTA |  |  |  |  |
| HO | Heme Oxygenase | F:TCCTCCTCCTCCAGCTCTATG | 150 | 116.1 | 62 | XM_014181218.1 |
|  |  | R:ACAAGTCCTTGGCCGCATTA |  |  |  |  |
| BLV | Biliverdin Reductase A | F:AAACAGATCCACCAGCCAGG | 150 | 106.6 | 60 | XM_014190443.1 |
|  |  | R:ACAGCCGACTTTAAGCAGCT |  |  |  |  |
| HEP | Hepcidin 1 | F:GCTGTTCCTTTCTCCGAGGTGC | 165 | 111.07 | 60 | NM_001140849.1 |
|  |  | R:GTGACAGCAGTTGCAGCACCA |  |  |  |  |
| FerritinM | ferritin, middle subunit-like | F:TATCACCACGATTGCGAAGC | 150 | 73.4 | 60 | XM_014190428.1 |
|  |  | R:CTCGTCGCTGTTCTCCTTGA |  |  |  |  |
| CBPBG | Coproporphyrinogen Oxidase | F: TCACCGATGCCAATTTCAGCT | 200 | 111.52 | 60 | Unpublished |
|  |  | R: GAGTGCGGTTCATGTCCCTC |  |  |  |  |
| Transferrin | Transferrin | F: GATGGGTCCAAGTGCAAAGC | 150 | 111.04 | 60 | XM_014190332.1 |
|  |  | R: TGCCCAAACTGGACCACTAC |  |  |  |  |
| Haptoglobin | Haptoglobin | F: GGCATGTAGGCAGAGAGCTT | 150 | 132.2 | 59 | XM_014182957.1 |
|  |  | R: GGAGAATGTGTGCTTTGGGG |  |  |  |  |
| Cathepsin A | Cathelicidin 1 | F: CAAGAAGAAGCCAAGCCAGA | 108 | 111.14 | 60 | AY728901.1 |
|  |  | R: GACCAATTAAGGAGCCACCC |  |  |  |  |
| Cathepsin B | Cathelicidin 2 | F: CTGTAAGGTTGAGCTTCCCC | 105 | 107.09 | 60 | XM_014140493.1 |
|  |  | R: TGCACATCATTCGTTTCCCA |  |  |  |  |
| Hepcidin | Hepcidin 1 | F: GCTGTTCCTTTCTCCGAGGTGC | 165 | 89.48 | 62 | NM_001140849.1 |
|  |  | R: GTGACAGCAGTTGCAGCACCA |  |  |  |  |
| IFNγ | Interferon Gamma | F: CTAAAGAAGGACAACCGCAG | 159 | 107 | 60 | AJ841811.1 |
|  |  | R: CACCGTTAGAGGGAGAAATG |  |  |  |  |
| MX1 | myxovirus resistance 1 | F: TGCAGCTGGGAAGCAAACT | 71 | 89.98 | 60 | NM_001123693.1 |
|  |  | R: CAACGTTTGGCTGATCAGATTC |  |  |  |  |
| Gig2 | interferon-inducible protein Gig2-like | F: GATGTTTCATGGCTGCTCAA | 119 | 107.4 | 60 | BT044022.1 |
|  |  | R: CTTTTCGGATGTCCCGACTA |  |  |  |  |
| WB117 | Infectious pancreatic necrosis virus, protein VP2 | F: GCGGTTCGACTTCATTCTACA | 100 | 116.7 | 60 | U48225.1 |
|  |  | R: GAGCTTGTCACGGAGACCAC |  |  |  |  |
|  |  | P: CTTGGGCT |  |  |  |  |

**Supplementary Table 1**.- Primers used for qRT-PCR.
